# Baseline disease duration of chronic spontaneous urticaria participants in phase III clinical trials

**DOI:** 10.1186/s13223-026-01026-0

**Published:** 2026-03-25

**Authors:** Sana Gupta, Vinesh Rao, Grace Xiong, Mohannad Abu-Hilal

**Affiliations:** 1 Faculty of Medicine, University of Ottawa, Ottawa, ON Canada; 2 Faculty of Medicine, University of Alberta, Edmonton, AB Canada; 3 Michael G. DeGroote School of Medicine, McMaster University, Hamilton, ON Canada; 4 Division of Dermatology, McMaster University, 206 Locke Street South, Hamilton, ON L8P 4B4 Canada

**Keywords:** Chronic spontaneous urticaria, Clinical trials

## Abstract

Chronic spontaneous urticaria often follows a prolonged and variable course, yet baseline disease duration is inconsistently reported in clinical trials, limiting interpretation of treatment outcomes. Accordingly, this systematic review aimed to evaluate baseline disease duration and reporting practices in phase-III trials for chronic spontaneous urticaria. Of 36 trials identified, only 16 (44.4%) reported baseline disease duration (mean: 5.30 ± 1.81 years). Differences in average disease duration were observed by publication date and intervention of interest. Definitions of disease duration were frequently unspecified, and no trials examined efficacy stratified by disease duration. This review ultimately highlights a gap in disease duration reporting, underscoring the need for standardization and transparency in clinical trial design.

**Supplementary Information:**

The online version contains supplementary material available at 10.1186/s13223-026-01026-0.

## Main text

Chronic spontaneous urticaria (CSU) is an inflammatory skin disease characterized by recurrent wheals or angioedema persisting beyond six weeks [1]. CSU often exhibits a variable clinical course, with symptomatic exacerbation and recurrence over time [2]. Disease duration, which is often variably defined across the literature as time to symptom onset or time to formal diagnosis, presents important implications for prognostic predictions, outcome generalizability and timing of biologic therapy, with longer durations demonstrating increased severity and reduced responsiveness to treatments [2]. However, it remains inconsistently characterized at baseline across CSU trials. This systematic review accordingly aims to evaluate baseline disease duration and reporting practices in phase-III CSU trials.

MEDLINE and Embase were searched from inception to October 2025. English-language phase-III trials investigating any treatment for CSU were included; abstracts, secondary analyses, and trials without disease duration reporting were excluded (Figure S1). Participant characteristics were summarized descriptively, and disease durations were pooled by calculating a weighted average across trials reporting mean CSU durations at baseline. Risk of bias, assessed using Cochrane ROB 2.0, was low in 14 trials and moderate in 2 trials (Figure S2).

Of 36 unique phase-III trials identified, 16 (44.4%) reporting mean baseline CSU duration were included. These trials comprised 3980 patients (68.5% female; mean age: 42.3 years) (Table 1). Mean body mass index (BMI) was 27.27. At baseline, participants had a mean weekly Urticaria Activity Score (UAS7) of 30.0 and weekly Itch Severity Score (ISS7) of 14.8. Angioedema was present in 44.4% (*n* = 1,493/3,362) of patients.

**Table 1:** Clinical and study characteristics of the included studies

Clinical characteristics (*n* = 3980)	*N* (%)
Age (y), mean	42.3
Sex, female	2728 (68.5)
Race/ethnicity (*n* = 3686) White Asian Black or African American Other	2115 (57.4) 1428 (38.7) 115 (3.1) 28 (0.8)
BMI, mean (*n* = 3629)	27.3
Disease duration (y), mean	5.3
Baseline UAS7, mean (*n* = 3560)	30.0
Baseline ISS7, mean (*n* = 2416)	14.79
Angioedema present (*n* = 3362)	1493 (44.4)
Intervention (*n* = 2902) Omalizumab Remibrutinib Omalizumab biosimilar Rupatadine Dupilumab Vitamin D3 Ligelizumab Cetirizine Fumaria syrup Methotrexate	1401 (48.2) 613 (21.1) 374 (12.9) 138 (4.8) 124 (4.3) 69 (2.4) 66 (2.3) 39 (1.3) 39 (1.3) 39 (1.3)
Study characteristics (*n* = 16)	N (%)
Blinding Double-blind Single-blind Open-label	14 (87.5) 1 (6.3) 1 (6.3)
Study population Adult only Mixed-age	6 (37.5) 10 (62.5)
Funding source Industry Academic Government	13 (81.3) 2 (12.5) 1 (6.3)

BMI: Body mass index; ISS7: weekly Itch severity score; UAS7: weekly Urticaria activity score.

Average baseline CSU duration across trials was 5.30 ± 1.81 years. Definitions of disease duration (time since diagnosis versus time since symptom onset) were frequently unspecified. Among 8 trials reporting ranges, disease duration spanned from 0 to 66.4 years. Participant-level distributions were unavailable, limiting assessment of how many had shorter-standing (< 1 year) versus longer-standing CSU (> 5 years) [3]. Dupilumab users reported the longest mean disease duration (7.18 years) compared to omalizumab (5.42 years) and remibrutinib (5.97 years) users (Table 2). Studies published after 2020 reported shorter average CSU duration (4.82 years) than earlier trials (6.00 years). No trials analyzed efficacy by CSU duration.

**Table 2:** Disease duration comparisons between trials (*n* = 16)

Disease duration (years)	Mean ± SD
Overall	5.30 ± 1.81
By drug class Dupilumab Remibrutinib Vitamin D3 Omalizumab Fumaria syrup* Methotrexate* Ligelizumab* Cetirizine* Rupatadine* Omalizumab biosimilar	7.18 ± 2.40 5.97 ± 1.06 5.45 ± 1.68 5.42 ± 2.07 5.10 4.90 4.80 4.70 3.58 3.08 ± 0.67
By publication date Before 2020 After 2020	6.00 ± 1.63 4.82 ± 1.83

*Single-trial data available for drug class.

This review highlights a gap in reporting of participant baseline disease duration before initiating treatment among phase-III CSU trials, with only sixteen of 36 identified CSU trials (44.4%) providing such data. Despite generally robust methodologies, trials lacked clear definitions of CSU duration, limiting accurate assessment of disease burden. While CSU involves symptom persistence beyond six weeks, average disease duration at enrollment exceeded five years, reflecting chronic, often refractory disease among patients accessing advanced therapies. Although not a factor in recruitment strategies, dupilumab trials generally tended to enroll patient cohorts with longer-standing disease, possibly reflecting a more treatment-refractory population, which may explain its lower observed efficacy compared with other agents. Given the more recent approval of dupilumab for CSU, participants may have previously failed multiple therapies including older agents such as omalizumab prior to enrollment, thus contributing to longer apparent disease duration at baseline. Baseline disease durations nevertheless ranged from new diagnosis to over six decades, underscoring heterogeneity among trials. Longer-standing CSU, particularly beyond 10 years, has been associated with greater recurrence and angioedema [4]. This variability may limit cross-trial comparability and assessment of duration-dependent differences in treatment effect. Evolving clinical practices, including earlier referrals, may further explain shorter disease durations in recent trials. Type I (IgE-mediated) and type IIb (autoantibody-mediated) autoimmune mechanisms have been implicated in CSU pathogenesis, with type IIb mechanisms demonstrating severe, longer-lasting disease and diminished responsiveness to antihistamines and omalizumab [5]. Disease duration may therefore act as a clinical surrogate for immunopathologic subtypes, reinforcing its relevance in trial design.

Overall, baseline disease duration is underreported in phase-III CSU trials, despite its clinical relevance. Enrolled populations generally had longstanding disease, but with substantial variability and no duration-stratified efficacy analyses, limiting interpretability and cross-trial comparability. Limitations of this review include methodological heterogeneity and lack of specific participant-level data. Alongside participant demographics, standardized reporting of baseline disease duration is needed to improve outcome interpretation and inform individualized management.

## Supplementary Information

Below is the link to the electronic supplementary material.

Supplementary Material 1

## Data Availability

The data that support the findings of this study are available from the corresponding author upon reasonable request.

## Abbreviations

BMI: Body mass index
CSU: Chronic spontaneous urticaria
ISS7: Weekly Itch Severity Score
UAS7: Weekly Urticaria Activity Score
ROB: Risk of bias

## References

[1] Lee R, Bernstein JA. Chronic spontaneous urticaria and chronic inducible urticaria. J Allergy Clin Immunol. 2025;156(3):546–56. 10.1016/j.jaci.2025.05.019. 40451490 10.1016/j.jaci.2025.05.019

[2] Armstrong AW, Soong W, Bernstein JA. Chronic spontaneous urticaria: How to measure it and the need to define treatment success. Dermatol Ther (Heidelb). 2023;13(8):1629–46. 10.1007/s13555-023-00955-7. 37354293 10.1007/s13555-023-00955-7 PMC10366057

[3] Balp MM, Halliday AC, Severin T, et al. Clinical remission of chronic spontaneous urticaria (CSU): A targeted literature review. Dermatol Ther (Heidelb). 2022;12(1):15–27. 10.1007/s13555-021-00641-6. 34807372 10.1007/s13555-021-00641-6 PMC8776966

[4] Stepaniuk P, Kan M, Kanani A. Natural history, prognostic factors and patient perceived response to treatment in chronic spontaneous urticaria. Allergy Asthma Clin Immunol. 2020;16(1):63. 10.1186/s13223-020-00459-5. 32834828 10.1186/s13223-020-00459-5 PMC7371813

[5] Lang DM, Sheikh J, Joshi S, Bernstein JA. Endotypes, phenotypes, and biomarkers in chronic spontaneous urticaria. Annals Allergy Asthma Immunol. 2024;134(4):408–e4173. 10.1016/j.anai.2024.10.026. 10.1016/j.anai.2024.10.026 39490777

