# Supplementary material for: Baseline disease duration of chronic spontaneous urticaria participants in phase III clinical trials
Source: Allergy Asthma Clin Immunol. 2026 Mar 25;22:26. doi: 10.1186/s13223-026-01026-0 (PMC13137646; doi:10.1186/s13223-026-01026-0)

**Supplemental Material**

Gupta, S., Rao, V., Xiong, G., et al. Disease Duration of Chronic Spontaneous Urticaria Participants in Phase III Clinical Trials

**Figure S1.** PRISMA flow diagram of literature screening.
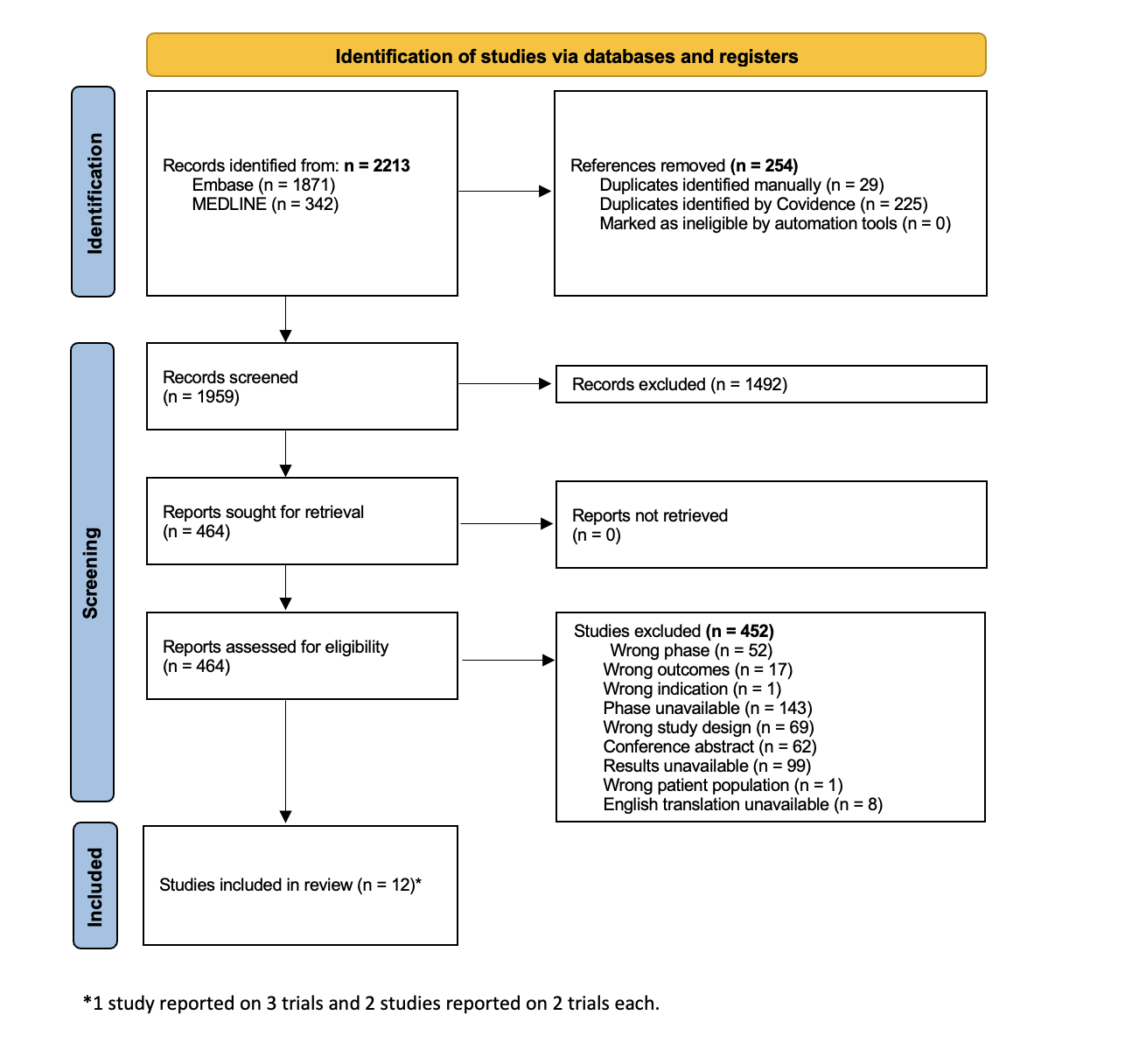


**Figure S2:** Cochrane ROB 2.0 assessment of included trials (n=16).


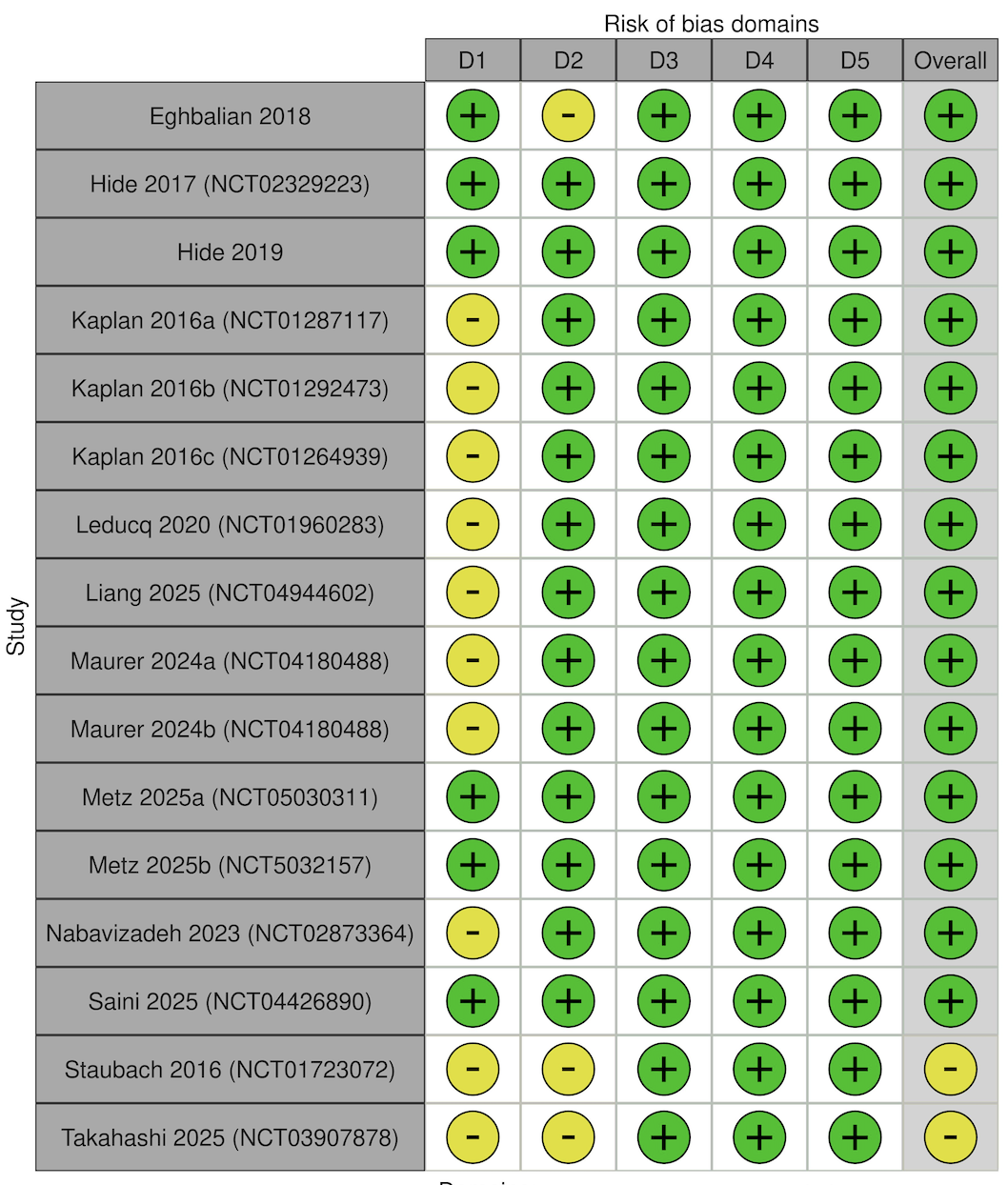

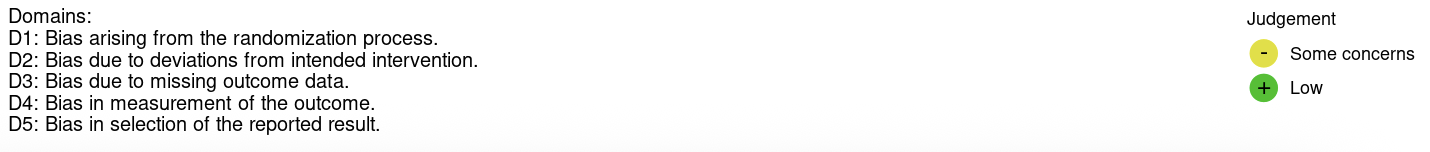

Supplement: Supplementary file 1 — Supplementary Material 1 [file 13223_2026_1026_MOESM1_ESM.docx]
